# Characterization of 46 patient-specific *BCR-ABL1* fusions and detection of SNPs upstream and downstream the breakpoints in chronic myeloid leukemia using next generation sequencing

**DOI:** 10.1186/s12943-015-0363-8

**Published:** 2015-04-18

**Authors:** Jana Linhartova, Lenka Hovorkova, Simona Soverini, Adela Benesova, Monika Jaruskova, Hana Klamova, Jan Zuna, Katerina Machova Polakova

**Affiliations:** Institute of Hematology and Blood Transfusion, Prague, Czech Republic; CLIP, Department of Paediatric Haematology and Oncology, 2nd Faculty of Medicine, Charles University Prague and University Hospital Motol, Prague, Czech Republic; Department of Experimental, Diagnostic and Specialty Medicine, Institute of Hematology “L. e A. Seragnoli”, University of Bologna, Bologna, Italy; Institute of Clinical and Experimental Hematology of First Faculty of Medicine and Institute of Hematology and Blood Transfusion, Charles University, Prague, Czech Republic

**Keywords:** BCR-ABL, Cancer, MRD, CML, SNP, NGS

## Abstract

In chronic myeloid leukemia, the identification of individual *BCR-ABL1* fusions is required for the development of personalized medicine approach for minimal residual disease monitoring at the DNA level. Next generation sequencing (NGS) of amplicons larger than 1000 bp simplified and accelerated a process of characterization of patient-specific *BCR-ABL1* genomic fusions. NGS of large regions upstream and downstream the individual breakpoints in *BCR* and *ABL1* genes, respectively, also provided information about the sequence variants such are single nucleotide polymorphisms.

## Background

*BCR-ABL1* transcript level monitoring is a crucial part of therapy response evaluation in patients with chronic myeloid leukemia (CML). Molecular response (MR) in CML is expressed in terms of log reduction in *BCR-ABL1* transcript levels from a standardized baseline on an international scale [1]. An increasing number of chronic phase CML patients treated with tyrosine kinase inhibitors (TKIs) achieve sustained deep MR, which has led to the initialization of TKI discontinuation studies [2,3]. Although existing results showing that permanent TKI discontinuation is feasible in a proportion of patients, more than half experience molecular relapse. More sensitive MRD detection might allow to more accurately stratifying patients according to the depth of response, hence to the greater or smaller likelihood of relapse after discontinuation. It was recently shown that MRD could be detected at DNA level despite patients were in stable MR with undetectable *BCR-ABL1* transcripts [4,5]. It is supposed that quantification of *BCR-ABL1* gene would be useful for MRD monitoring in patients with therapy cessation.

At the genomic level the *BCR-ABL1* fusion is unique to each CML patient, since breakpoints are scattered within intron 13 (718 bp) or 14 (2128 bp) of *BCR* and within intron 1 of *ABL1* (140 Kbp). The exact genomic sequence of the *BCR-ABL1* fusion must therefore be characterized to design a personalized real-time polymerase chain reaction (PCR) assay. The sensitivity of personalized real-time PCR assay quantifying clone-specific fusions was reported to be down to less than 10^−5^ (depending on the amount of DNA entering the reaction) when nested PCR was used [6,7]. As amplification of large regions (˃1 Kbp) is needed, the characterization of *BCR-ABL1* genomic fusions using conventional sequencing has been a challenging task [8-10]. With the aim to characterize patient-specific *BCR-ABL1* fusions, we set up a strategy based on the generation of large amplicons by multiplex Long-Range PCR (mLR-PCR) and on subsequent ultra-wide sequencing using NGS technology. NGS provided sequences of large regions upstream and downstream the *BCR-ABL1* fusions, allowing us to assess the frequency of annotated single nucleotide polymorphisms (SNPs), which could be associated with the fusion gene formation.

## Findings

### Next generation sequencing of large amplicons carrying *BCR-ABL1* fusions

The patient cohort consisted of 24 males and 22 females with CML. Two *BCR-ABL1* positive cell lines K562 and JURL-MK were included as controls of *BCR-ABL1* fusion identification. Twelve healthy individuals were used as controls for SNP detection in the *BCR* region from exon 13 to exon 15.

**Figure 1:**
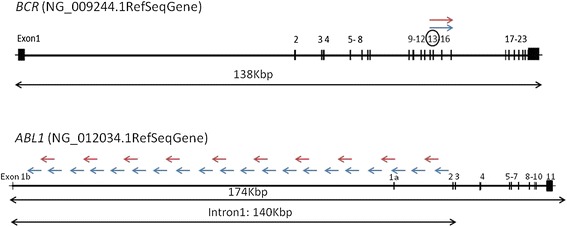
Schematic localization of primers. Forward primers hybridizing in exon 13 of the *BCR* gene are symbolized by arrows pointing to the right and reverse primers spanning the intron 1 of gene *ABL1* are symbolized by arrows pointing to the left. Colors of arrows for each gene illustrate two-rounds of multiplex LR-PCR. LR-PCR products were obtained in 25/48 cases from the first round PCR using 1 forward primer (red color) localized in *BCR* exon 13 and 10 reverse primers (red color) spanning the whole *ABL1* intron 1 [9]. LR-PCR products were obtained in the remaining 23/48 cases from the second round PCR with 1 forward primer (blue color) hybridizing to sequence of *BCR* exon 13 and 20 reverse primers (blue colors) hybridizing to ABL1 intron 1 [7,10].

DNA was isolated from total leukocytes of peripheral blood at the time of diagnosis ensuring high amount of *BCR-ABL1* DNA present in the sample. Two-round mLR-PCR was performed with HiFi Accuprime Taq DNA polymerase (Invitrogen) enabling amplification of large regions. The amplification strategy is presented in Figure 1. In the first round PCR with one primer in *BCR* exon 13 and 10 primers in *ABL1* intron 1 [9], the long PCR products of 29 patients were obtained. In the second round PCR with one primer in *BCR* exon 13 and 20 primers in *ABL1* intron 1 [10-12] products were obtained in the remaining cases. Median length of long PCR products was 5 Kbp (range 1-12 Kbp). Subsequently, rapid library was prepared for 454 NGS technology according to the manufacturer’s instructions and was run on a GS Junior instrument (Roche Diagnostics). NextGene software (Softgenetics) and reference sequences NG_009244.1 (*BCR*) and NG_012034.1 (*ABL1*) were used for sequence analysis.

### Patient-specific *BCR-ABL1* fusion characterization

**Figure 2:**
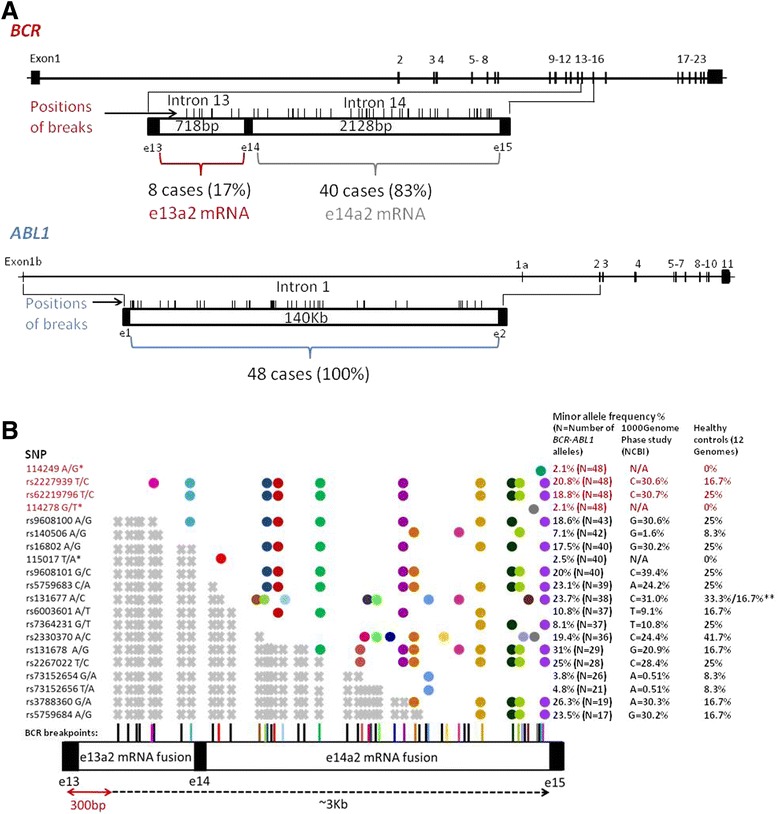
**A**. Breakpoint traces in *BCR* intron 13 and intron 14 and in *ABL1* intron 1. **B**. SNPs detected in individual *BCR-ABL1* alleles upstream the BCR breakpoints. Dots show detected SNPs; colors of dots correspond to each patient in whom SNPs were detected; colors of BCR breakpoints indicate each patient in whom SNPs were detected; BCR breakpoints shown in black represent patients in whom SNPs were not detected; *novel SNPs (numbers indicate position in ref. seq. NG_009244.1) **minor allele in a homozygous form; BCR region sequenced in all patients; SNPs whose rs code is in red font are localized within this region. SNPs not detectable in patients because of their localization beyond the breakpoints. Twelve healthy donor samples (controls) were used as controls for SNP detection in the *BCR* region from exon 13 to exon 15.

Breakpoints in *BCR* were located in intron 13 in 8/46 patients, carrying the e13a2 mRNA fusion, and in intron 14 in 38/46 patients and in both cell lines, carrying the e14a2 mRNA fusion (Figure 2A). All *ABL1* breakpoints were spread within intron 1 (Figure 2A), which is in accordance with a previous study [7]. Sequences of characterized breakpoints are provided in the NCBI Nucleotide database (Accession numbers – KR091980-KR092025).

### SNPs detection upstream and downstream from the *BCR-ABL1* fusions

Downstream the individual breakpoints in *ABL1*, 56 annotated SNPs were detected in 15/46 patients (median 3 SNPs/patient, range 1–11). Only 10 of them recurred in two patients, whereas the remaining 46 were detected only once. This heterogeneity in SNP detection was caused by the extreme length of intron 1 (Figure 1, Figure 2A), where the breakpoints were spread, so that the individual *ABL1* regions sequenced could not overlap.

Upstream the individual BCR breakpoints, 17 annotated SNPs and 3 novel variants were found altogether in 27/48 cases (56%) (Figure 2B). The dbSNP assigned accessions of newly detected variants (putative SNPs) are as follows:

- NG_009244.1:g.114249A > G|rs527236141|ss1227131697;NG_009244.1:g.114278G > T|rs527236142|ss1227132188;NG_009244.1:g.115017 T > A|rs527236143|ss1227132282.

The 300 bp region downstream of primers hybridizing to BCR exon 13 was sequenced in all cases (Figure 2B). Four SNPs were identified within this region altogether in 9% of patients with e13a2 mRNA fusion and in 12% of patients with e14a2 mRNA. Other 16 SNPs were detected almost exclusively in patients with e14a2 mRNA fusion, which is in accordance with the fact, that the BCR region sequenced in these patients was larger than in the patients with e13a2 mRNA. The minor allele frequency among the *BCR-ABL1* alleles analyzed corresponds to that found in the 1000 GenomePhase Population and in the cohort, although small, of healthy donors (Figure 2B).

## Conclusion

Although increasing number of studies has been published in last few years [6-16], analysis of *BCR-ABL1* at the DNA level has been limited and difficult so far. Our mLR-PCR-NGS approach streamlined the laboratory workflow for patient-specific *BCR-ABL1* fusion characterization allowing personalizing real-time PCR assays for patients with MRD. The large collection of 46 *BCR-ABL1* fusion sequences was obtained in the short time and is at disposal in the NCBI Nucleotide database. Moreover, the NGS enabled to detect SNPs upstream and downstream the patient-specific breakpoints of the *BCR-ABL1* gene. NGS of this region in large series of patients has the potential to provide insights on the association between particular SNPs or haplotypes and CML. Although we describe the method on *BCR-ABL1* in patients with CML*,* the strategy can be used in the same manner as a base for DNA-based MRD monitoring in BCR-ABL1 positive acute lymphoblastic leukemia (ALL), where significant differences between MRD levels measured by *BCR-ABL1* expression and Ig/TCR DNA-based approach were described [17]. Moreover identical approach can be used for breakpoints localization and sequence features calling in other large fusion genes associated with various cancers, e.g. *TEL/ABL1* (atypical CML or ALL) [18], *TEL/AML1* (acute lymphoid leukemia) [19], *TMPRSS2/ERG* (prostate cancer) [20] or *EML4/ALK* (non-small cell lung cancer) [21].
